# Environmental exposomics and lung cancer risk assessment in the Philadelphia metropolitan area using ZIP code–level hazard indices

**DOI:** 10.1007/s11356-021-12884-z

**Published:** 2021-02-21

**Authors:** Thomas P. McKeon, Wei-Ting Hwang, Zhuoran Ding, Vicky Tam, Paul Wileyto, Karen Glanz, Trevor M. Penning

**Affiliations:** 1 Center of Excellence in Environmental Toxicology, University of Pennsylvania, Philadelphia, PA 19104 USA; 2 Departments of Systems Pharmacology & Translational Therapeutics, University of Pennsylvania, 1315, BRBII/III, 421 Curie Blvd, Philadelphia, PA 19104 USA; 3 Biostatistics, Epidemiology & Informatics, University of Pennsylvania, Philadelphia, PA 19104 USA; 4 Abramson Cancer Center, University of Pennsylvania, Philadelphia, PA 19104 USA; 5 Cartographic Modeling Laboratory Perelman School of Medicine, University of Pennsylvania, Philadelphia, PA 19104 USA

**Keywords:** Carcinogens, Air pollution, Geospatial modeling, Toxic release inventory, Exposome, Hazard index

## Abstract

To illustrate methods for assessing environmental exposures associated with lung cancer risk, we investigated anthropogenic based air pollutant data in a major metropolitan area using United States-Environmental Protection Agency (US-EPA) Toxic Release Inventory (TRI) (1987–2017), and PM_2.5_ (1998–2016) and NO_2_ (1996–2012) concentrations from NASA satellite data. We studied chemicals reported according to the following five exposome features: (1) International Agency for Research on Cancer (IARC) cancer grouping; (2) priority EPA polycyclic aromatic hydrocarbons (PAHs); (3) component of diesel exhaust; (4) status as a volatile organic compound (VOC); and (5) evidence of lung carcinogenesis. Published articles from PubChem were tallied for occurrences of 10 key characteristics of cancer-causing agents on those chemicals. Zone Improvement Plan (ZIP) codes with higher exposures were identified in two ways: (1) combined mean exposure from all features, and (2) hazard index derived through a multi-step multi-criteria decision analysis (MMCDA) process. VOCs, IARC Group 1 carcinogens consisted 82.3% and 11.5% of the reported TRI emissions, respectively. ZIP codes along major highways tended to have greater exposure. The MMCDA approach yielded hazard indices based on imputed toxicity, occurrence, and persistence for risk assessment. Despite many studies describing environmental exposures and lung cancer risk, this study develops a method to integrate these exposures into population-based exposure estimates that could be incorporated into future lung cancer screening trials and benefit public health surveillance of lung cancer incidence. Our methodology may be applied to probe other hazardous exposures for other cancers.

**Supplementary Information:**

The online version contains supplementary material available at 10.1007/s11356-021-12884-z.

## Introduction

In the United States, lung cancer is the leading cancer killer in both men and women (Siegel et al. 2020). An estimated 135,720 Americans died of lung cancer in 2019, accounting for 22% of all cancer deaths (American Lung Association (ALA) 2020). The Philadelphia metropolitan area, part of the Greater Delaware Valley, has lung and bronchus cancer incidence rates of 75.8 per 100,000, higher than the national rate of 59.3 per 100,000 and Pennsylvania’s rate of 64.0 per 100,000 for the 2013 to 2017 timeframe (The Centers for Disease Control and Prevention (CDC) 2020; Pennsylvania Department of Health (EDDIE) 2020). Data from the National Lung Cancer Screening (LCS) Trial suggests that LCS identifies less than 50% of patients who will develop lung cancer and only 4% of individuals that are eligible for LCS seek low-dose computed tomography (Kramer et al. 2011; National Lung Screening Trial Research 2011a, National Lung Screening Trial Research 2011b). Developing methods to identify sub-populations at risk for developing lung cancer could improve the outcomes of lung cancer screening trials and inform “precision” lung cancer screening. More generally, precision public health refers to targeting valuable resources to the most vulnerable as defined by Dowell et al. (Dowell et al. 2016).

While smoking is the main cause of lung cancer, accounting for close to 85–90% of all lung cancer cases, there are other environmental risk factors that contribute to lung cancer (International Agency for Research on Cancer (IARC) 2016; Zakaria et al. 2017). These exposures likely cause lung cancer in never smokers and increase lung cancer risk in smokers (Corrales et al. 2020). For example, combined exposure to asbestos and cigarette smoke, or diesel exhaust and smoking history increases incidence of lung cancer over that associated with smoking alone (Eckel et al. 2016; Benbrahim-Tallaa et al. 2012; Garshick et al. 2006). In the concept of monitoring life-long exposure to carcinogens for cancer risk, the “exposome” was originally promoted by Christopher Wild (Wild, 2005) and has been embraced by others (Dennis et al. 2017; Rappaport et al. 2014). While measuring an individual’s exposome may not be possible, an alternative is to provide a population level assessment of hazardous exposures in the environment (Baldwin et al. 2013).

Air pollution is classified by the International Agency for Research on Cancer (IARC) as a Group 1 carcinogen (i.e., carcinogenic to humans) (IARC 2013) that accounts for more than 220,000 lung cancer deaths per year worldwide and shortens survival after diagnosis (Lelieveld et al. 2015; Loomis et al. 2013). In considering the causal effect of air pollution on lung cancer, it is necessary to consider the carcinogens present in this exposure source. For example, many polycyclic aromatic hydrocarbons (PAHs) (e.g., benzo[*a*]pyrene, benz[*a*]anthracene, dibenz[*a*]anthracene), routinely measured by the EPA (EPA 2008), have been classified by IARC (Khadhar et al. 2010). Air pollutants also include volatile organic compounds (VOCs) that are classified as either IARC Group 1 carcinogens (butadiene (IARC, 1999a), benzene (IARC, 2018), formaldehyde (IARC, 1999b)) or IARC Group 2B carcinogens (acetaldehyde) (Seitz and Stickel 2009). The products of diesel fuel combustion are another important contributor to air pollution and consist of a mixture of gases and fine particulates such as nitroarenes, known as diesel particulate matter (DPM) (Consonni et al. 2018; Gharibvand et al. 2017; Occupational Safety and Health Administration, 2020). Although diesel technology has improved to control for many of these harmful emissions, the speciation of individual nitroarenes and VOCs which are components of the mixture allows us to distinguish between known chemicals in this mixture that may be released independently of vehicular diesel exhaust, including light-duty cars/trucks and other industrial practices that may use off-road diesel-driven machinery (El-Bayoumy et al. 1989; Enya et al. 1997). Importantly, the nitroarenes include 3-nitobenzanthrone, one of the most mutagenic compounds identified in the Ames test, and 6-nitrochrysene, a potent tumorigen in the newborn mouse lung.

It is problematic that many chemicals present in the environment are unclassified by IARC or have not yet been evaluated by IARC. Unclassified status by IARC (i.e., Group 3) does not necessarily mean it is not carcinogenic to humans but merely that there is insufficient evidence to date. For example, among 695 chemicals currently listed under the EPA Toxic Release Inventory (TRI) Program (EPA, 2020a), 18.8% are unclassified or not evaluated by IARC. In the IARC classification, mode-of-action data has used a subjective analysis; by contrast, Smith et al. identified 10 key characteristics (KCs) of cancer-causing agents that contribute to carcinogenesis (Smith et al. 2016). Subsequent application of the 10 KCs determined their predictive value for chemicals classified by IARC and revealed strong evidence that multiple KCs for most Group 1 or 2A agents exist to support their classification and support the use of these KCs to capture carcinogenic risk of unknowns (Guyton et al. 2018).

The objective of this research is to illustrate a methodology to aggregate environmental exposures known to be associated with increased lung cancer risk. Using the Philadelphia region as an example, we applied geospatial approaches to (1) map hazardous chemicals in the areas of interest using publicly available air pollution data, (2) synthesize multiple hazardous chemicals into one summary measurement, and (3) identify ZIP codes with high exposure using a hazard index in order to identify populations which may benefit from increased LCS.

## Materials and methods

### Study region

The study area consists of 12 counties of the greater Delaware Valley—five in Pennsylvania (Bucks, Chester, Delaware, Montgomery, and Philadelphia Counties), six in New Jersey (Atlantic, Burlington Camden, Gloucester, Mercer, and Ocean Counties), and one in Delaware (New Castle County). County boundaries for the year 2018 and ZIP code boundaries for the year 2019 were sourced from the United States Census Bureau (Census, 2020).

Environmental Systems Research Institute (ESRI)’s geospatial software, ArcGIS, was used to create spatial layers of the study area. We extracted ZIP codes which have their geographic centroid within the 12 counties, resulting in 421 ZIP codes. The WGS84 coordinate system was defined for all layers to ensure geographic consistency.

### Data sources

#### Toxic Release Inventory data

EPA’s TRI program tracks the management of toxic chemicals by point source that may pose a threat to human health and the environment (Bulka et al. 2016). We downloaded TRI annual-reporting data for 1987 to 2017 from EPA’s *Data Mart* with information on point source, and the amount of chemical emissions (in pounds) released into either water, land, or air by each reporting year (EPA, 2020b). Given that our study is investigating air pollution, we only considered air emissions by summing fugitive air and stack air emissions into one combined value. The total number of active TRI-reporting facilities varied over the years and peaked at 445 in 1990 and declined to 177 in 2017. We selected chemicals in the present analysis if they met any of the following exposomics features: (1) Is classified in IARC Groups 1 to 3 as a carcinogen. Their grouping is as follows: Group 1: carcinogenic to humans; Group 2A: probably carcinogenic to humans; Group 2B: possibly carcinogenic to humans, and Group 3: not classifiable as to its carcinogenicity to humans (IARC 2020); (2) Is one of the EPA 16 priority PAHs, as a surrogate marker of exposure to carcinogens (Hussar et al. 2012); (3) Is a component of diesel exhaust (Steiner et al. 2016); (4) Is a VOC based on 113 chemicals listed in EPA’s parameter code for VOCs (EPA 2020c); and (5) Is a lung carcinogen with limited or sufficient evidence of lung carcinogenesis (Cogliano et al. 2011). In total, 201 TRI chemicals met these criteria and were selected.

#### NASA satellite data

Publicly available satellite-derived grids were sourced from NASA for years when data were available (NASA 2020). As a measure of fine particulates that may be impregnated with carcinogens, annual global surface concentrations for PM_2.5_ in micrograms per cubic meter at 1-kilometer (km) resolution were available for years 1998 to 2016 (NASA 2018). As a surrogate for traffic, global 3-year running means for NO_2_ concentrations in parts per billion at 10-km resolution were available for years 1996 to 2012 (NASA 2017).

### Mapping and combining cumulative exposure

TRI facility locations and their reported air emissions for all available years (1987 to 2017) provided data to generate kernel density raster-level values with a magnitude-per-1-km resolution area for each of the five TRI exposomic features using the ArcGIS *Spatial Analyst* toolbox. NASA satellite data for both PM_2.5_ and NO_2_ were projected onto the Philadelphia study region as raster values for all available years. We used *Raster Calculator*, a built-in ArcGIS tool, to generate cumulative exposure layers for each of the NASA and TRI exposomic features by summing the grouped kernel density values across all available years. We presented maps of the density values or “heat-maps” with a color gradient ranging from a low to high emissions. Again, using *Raster Calculator*, we incorporated the cumulative exposure layers of each NASA and TRI features into a single combined mean exposure layer by summing the values and dividing by the number of incorporated layers. Thirty years of TRI features, 18 years of NASA PM_2.5_ data, and 16 years for the NASA NO_2_ data made up the combined incorporated layers. The resulting raster layer provides a gradient visualization of low to high mean combined mean exposure across all exposure sources studied.

### Multi-step multi-criteria decision analysis

This multi-step multi-criteria decision analysis (MMCDA) is a risk assessment framework modified from EPA’s existing multiple-criteria decision analysis (MCDA) framework (EPA 2015). The MCDA had previously been used for hazard evaluation of chemicals found in hydraulic fracturing fluids using “toxicity,” “persistence,” and “occurrence” criteria (Yost et al. 2017; Mitchell et al. 2013; Huang et al. 2011). The goal of the modified framework is to quantify and rank the risk of exposure to chemical mixtures emitted into the air or the environment. This approach is a way to integrate multiple exposures into one aggregate index for population-based risk estimates based on assessing specific air pollutant chemicals to derive a hazard index. This approach allows for the scoring of chemical toxicity (in some instances based on a literature search to weight the presence of the 10 key characteristics of a chemical carcinogen), persistence (volatile or non-volatile), and occurrence (amount released over time/versus the total amount of emission over time). The MMCDA permits the development of a point system to derive a hazard index by considering the following 3 criteria: (1) toxicity of a chemical, (2) persistence of a chemical, and (3) occurrence of a chemical in the geographical area unit under study. The TRI chemicals selected for MMCDA in this study are those described in the “Data sources” section.

#### Chemical toxicity score

The toxicity criterion consists of two sub-criteria. The first sub-criterion is based on the IARC groupings. A chemical receives a sub-criterion score of 1 point if it is in IARC Group 3, 2 points if it is in IARC Group 2B, 3 points if it is in IARC Group 2A, 4 points if it is in IARC Group 1, and 0 points if it has not been evaluated by IARC. The second sub-criterion is based on the amount of evidence published in the literature regarding a chemical’s carcinogenicity. Using PubChem, a publicly available online chemical database (PubChem 2020), we downloaded the title, abstract, and author information for all publications (before April 2019) associated with each selected chemical and tallied the total number of mentions of the following 10 key characteristics (KCs) of chemical carcinogenicity: (1) electrophilic or can be metabolically activated; (2) genotoxic; (3) alters DNA repair or causes genomic instability; (4) induces epigenetic alterations; (5) induces oxidative stress; (6) induces chronic inflammation; (7) is immunosuppressive; (8) modulates receptor-mediated effects; (9) causes immortalization; and (10) alters cell proliferation, cell death, or nutrient supply. In addition, for each chemical, we tallied the total number of times when the words “human,” “animal,” “tumor” (HATs) appeared across all publications. IARC weighs human subject and tumorigenicity findings heavily in its cancer risk assessment (IARC, 2020). The HATs score is the total number of mentions across all publications and was used to weigh the KCs.

Increased mention of the KCs and HATs likely indicates greater evidence of carcinogenicity, thus we assigned points to chemicals according to the distribution of mentions for KCs or HATs from all chemicals considered. Specifically, 1 point if a chemical belongs to the lowest 25%tile of the distribution, 2 points if it is within the 25% to 50%tiles, 3 points if it is within the 50% to 75%tiles, and 4 points if it is within the upper 25%tile of the distribution. Points were assigned separately for the KC and HATs quartiles but the higher point of the two was taken as the second sub-criterion score for that chemical. The two sub-criteria scores are then summed to yield the raw toxicity score. The maximum value for the raw toxicity score is 8. As an example, the chemical styrene is classified by IARC as Group 2A with 3 points (first sub-criteria). Styrene is also in the 2nd quartile for HATs distribution (2 points) and 3rd quartile for KCs distribution (3 points), the higher of which is 3 points as the second sub-criteria score. The raw toxicity score for styrene is 6 points by adding 3 points (the first sub-criterion) with 3 points for KCs (the second sub-criterion), the maximum of either KC or HAT criteria.

#### Persistence score

The persistence criterion is based on whether the chemical is a VOC; since VOCs do not persist as long as non-VOCs, a VOC chemical receives a score of 0 point and a non-VOC chemical receives a score of 1 point. The criteria of persistence commonly used for EPA’s MCDA also consider vapor pressure. For air toxic exposure, we used only a binary measure to estimate persistence.

#### Rescale raw scores

The raw scores for each chemical’s toxicity and persistence were then rescaled by using the following formula: *S*_x_rescaled_ = (*S*_x_ − *S*_min_)/(*S*_max_ − *S*_min_), where *S*_x_ corresponds to the raw score for chemical x, *S*_max_ is the highest observed score in the set of chemicals, and *S*_min_ is the lowest observed score. *S*_x_rescaled_ is the rescaled score for chemical x ranging between 0 and 1.

#### Risk score

The final risk score for a chemical is created by summing the rescaled toxicity (0–1) and rescaled persistence scores (0–1) and ranges from 0 to 2 with the higher score indicating a higher risk. These scores serve as a relative ranking and a way of comparing risk across a set of chemicals before incorporating the occurrence of emissions to compute the final hazard index in the steps as described below.

#### Occurrence score

The occurrence score is calculated as the fraction of a chemical released (in pounds) to a geographical unit of interest such as ZIP codes out of the total amount released for the same chemical in all ZIP codes combined. If the focus is on the occurrences of chemicals within a different timeframe or a different geographic area, a subset of the TRI database can be selected to calculate the fractions.

#### Hazard index

Lastly, the final hazard index for each ZIP code was calculated by summing all the chemicals’ occurrence fractions for the ZIP code weighted by the risk score for the chemical. That is, hazard index for ZIP code *i* = sum__ j_ (fraction of chemical j released in ZIP code *i* relative to chemical *j* released in all ZIP codes) × (risk score for chemical *j*). As an example, suppose three chemicals X, Y, Z were released in ZIP code 08534. The risk scores for chemicals X, Y, and Z are 1.8, 0.9, and 0.5, respectively. If 200 pounds of X, 800 pounds of Y, and 600 pounds of Z were released in 08534, and 15,000 pounds of X, 10,000 pounds of Y, 20,000 pounds of Z released across all ZIP codes combined, then the hazard index for ZIP code 08534 would be: 1.8 × (200/15,000) + 0.9 × (800/10,000) + 0.5 × (600/20,000) = 0.111

## Results

### Emissions from Toxic Release Inventory

Annual TRI data from 1987 to 2017 reported the cumulative release of 268,054,248 lbs of air emissions for 110 out of the 201 chemicals that met one or more of the five exposomic features in the study area. These exposomic features were (1) IARC cancer grouping, (2) priority EPA PAHs, (3) component of diesel exhaust, (4) status as a VOC, and (5) evidence of lung carcinogenesis. Of these emissions, 11.5% (30,935,548 lbs) were from 16 unique chemicals that were classified as IARC Group 1; 2.1% (5,567,528 lbs) were from 8 unique chemicals that were classified as IARC Group 2A; 12.3% (33,054,547 lbs) were from 33 unique chemicals that were classified as IARC Group 2B; and 44.7% (119,894,567 lbs) were from 24 unique chemicals that were classified as IARC Group 3. Four chemicals on the EPA list of 16 priority PAH account for 0.4% (965,911 lbs), including 61,780 lbs reported as unspecified PAHs; and 2.8% (7,406,592 lbs) came from six chemicals listed as components of diesel exhaust. Most of the emissions, 82.3% (220,783,477 lbs) came from 44 unique VOC chemicals. Nine unique chemicals came from the list of limited to sufficient evidence of human lung carcinogenesis and accounted for 3.1% (8,309,602 lbs) of the total emissions.

### Mapping cumulative exposures by feature

**Fig. 1:**
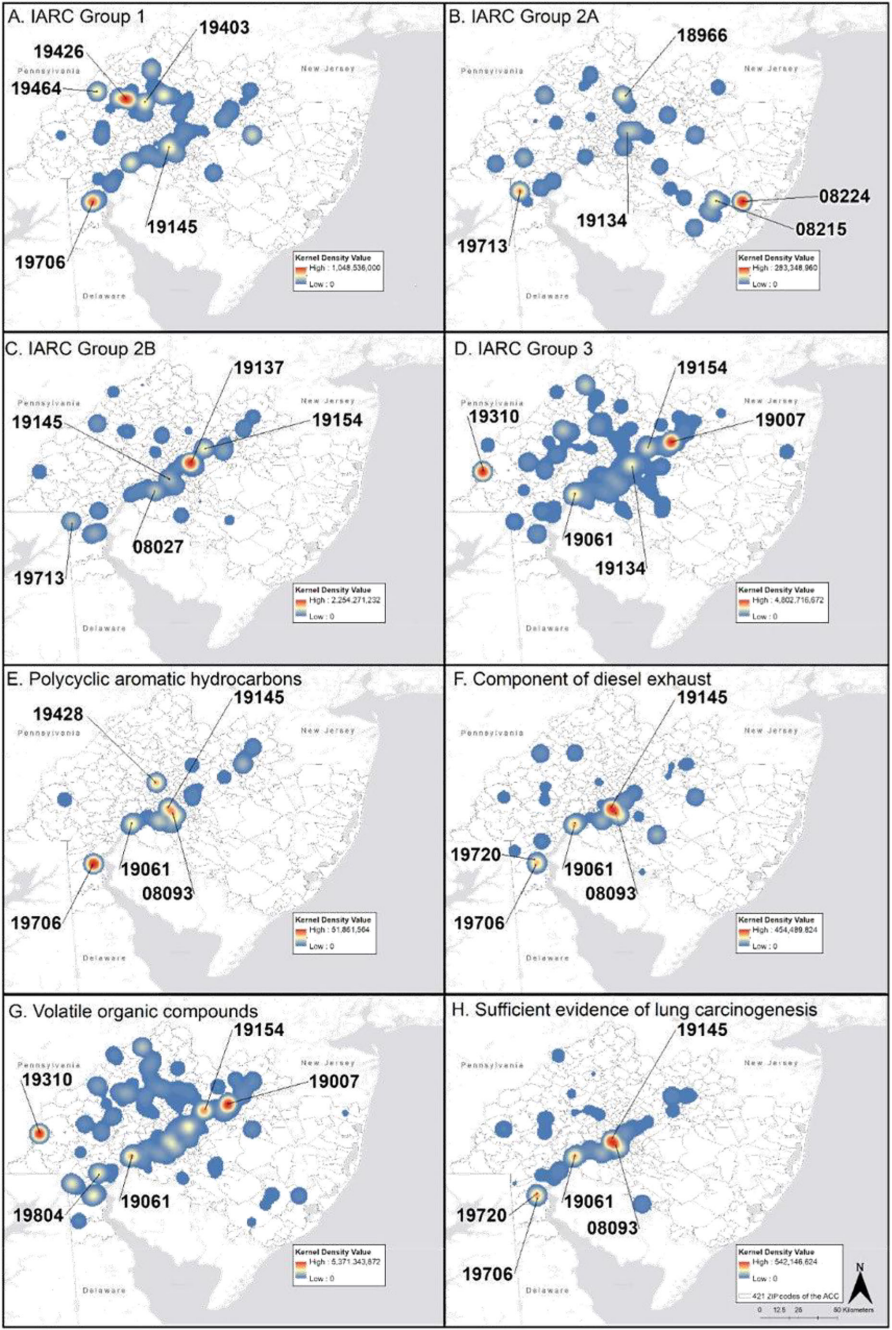
**a**–**h** Cumulative exposure of TRI exposomic features: **a** IARC Group 1; **b** IARC Group 2A; **c** IARC Group 2B; **d** IARC Group 3; **e** priority EPA PAHs; **f** component of diesel exhaust; **g** VOC status; and **h** evidence of lung carcinogenesis

Kernel density maps of the five exposure features are shown in Fig. 1 a–g. IARC Group 1 emissions were highest in 19426 (Collegeville, PA), 19706 (Delaware City, DE), 19403 (West Norriton, PA), 19464 (Pottsgrove, PA), and 19145 (Philadelphia Energy Solutions (PES) Oil Refinery Region). IARC Group 2A emissions were highest in 08224 (New Gretna, NJ), 08215 (Egg Harbor City, NJ), 19713 (Newark, DE), 18966 (Southampton, PA), and 19134 (Kensington/Port Richmond, PA). IARC Group 2B emissions were highest in 19137 (Bridesburg, Philadelphia), 19154 (Parkwood, PA), 08027 (Gibbstown, NJ), 19713 (Newark, DE), and 19145 (PES Oil Refinery Region). IARC Group 3 emissions were highest in 19007 (Bristol, PA), 19310 (Atglen, PA), 19061 (Marcus Hook, PA), 19154 (Parkwood, PA), and 19134 (Kensington/Port Richmond, PA). The cumulative emissions of chemicals classified as PAHs were highest in ZIP codes 19706 (Delaware City, DE), 19145 (PES Oil Refinery Region), 19428 (Conshohocken, PA), 19061 (Marcus Hook, PA), and 08093 (Westville, NJ). Of the 16 priority EPA PAHs, only naphthalene, phenanthrene, anthracene, and benzo[g,h,i] perylene were reported. However, the chemical grouping of “polycyclic aromatic compounds” was used to report a significant amount of PAH emissions, creating uncertainty as to which PAHs were released. Benzo[a]pyrene, the only PAH that is a Group 1 carcinogen, was not reported in this TRI dataset, but may have been included in the “polycyclic aromatic compounds” grouping. The highest cumulative emissions of chemicals classified as components of diesel exhaust emissions were found in 19145 (PES Oil Refinery Region), 19061 (Marcus Hook, PA), 08093, (Westville, NJ), 19706 (Delaware City, DE), and 19720 (New Castle, DE). All the classified toxicologically relevant components of diesel exhaust were detected except the nitroarenes. Forty-four VOCs were reported, and emissions were highest in 19007 (Bristol, PA), 19310 (Atglen, PA), 19154 (Parkwood, PA), 19061 (Marcus Hook, PA), and 19804 (Stanton, DE). The highest cumulative emissions of chemicals with sufficient or limited evidence of lung carcinogenesis were found in the same ZIP codes as diesel exhaust emissions.

#### NASA satellite imagery

**Fig. 2:**
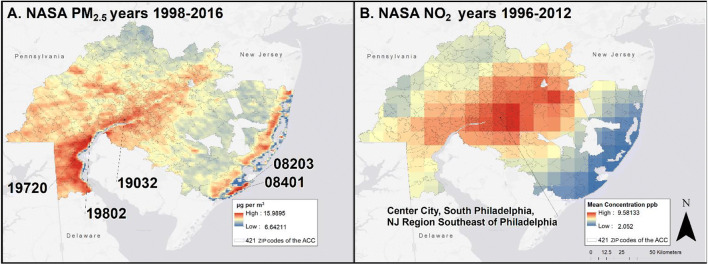
**a**, **b** NASA satellite-derived cumulative exposure for **a** PM_2.5_ (1998–2016) and **b** NO_2_ (1996–2012)

The cumulative emissions of exposures to the NASA data are shown in Fig. 2 a and b. Higher concentrations of PM_2.5_ were along the southern New Jersey shore and along the regions corresponding to major highways, as shown in Fig. 2 a. The cumulative NASA PM_2.5_ observations were highest in 19032 (Folcroft, PA), 19802 (Wilmington, DE), and 19720 (New Castle, DE). The cumulative NASA NO_2_ observations were only available in a 10-km resolution, and was not as precise as the NASA PM_2.5_ and made pinpointing exposure at a ZIP code level impossible. The highest NO_2_ levels were around Center City, Philadelphia, South Philadelphia, and the New Jersey region across the Delaware river from South Philadelphia and is shown in Fig. 2 b.

### Combined mean exposure by TRI features and NASA data

**Fig. 3:**
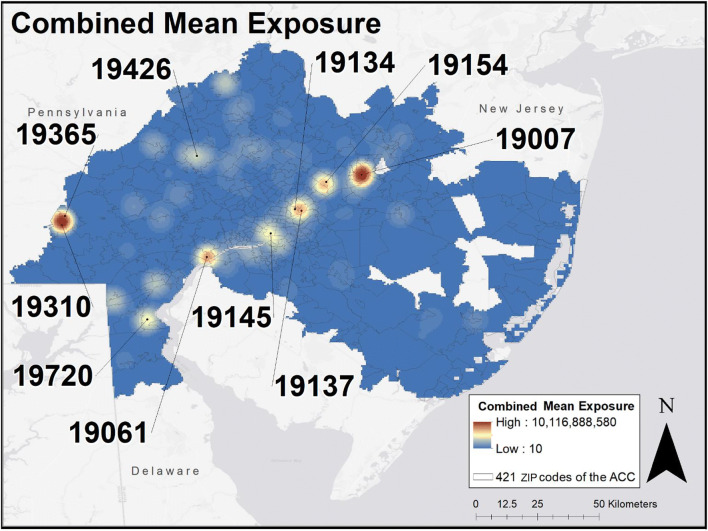
Combined mean exposure of TRI and NASA exposomic feature

The map of combined mean exposure that incorporates all the features is presented in Fig. 3. The greatest burden was found in the following ZIP codes: 19007 (Bristol, PA), 19310 (Atglen, PA), 19154 (Parkwood, PA), 19061 (Marcus Hook, PA), 19137 (Bridesburg, Philadelphia), 19426 (Collegeville, PA), 19365 (Parkesburg, PA), 19134 (Kensington/Port Richmond, PA), 19145 (PES Oil Refinery Region, PA), 19720 (New Castle, DE)

### Hazard index derived from MMCDA

Among 201 TRI chemicals selected, the rescaled toxicity score ranged from 0 (32 chemicals) to 1 (benzene, cadmium, chromium compounds, dioxin, ethylene oxide, formaldehyde, nickel compounds, phosphorus, trichloroethylene, vinyl chloride). The risk score (toxicity rescaled score + persistence rescaled score) ranged from 0 (28 chemicals) to 2 (cadmium, chromium compounds, dioxin, ethylene oxide, nickel compounds, phosphorus). Out of the 201 selected chemicals, 55.2% are VOCs. Each chemical’s exposomic feature classification, KC and HATs quartile ranking, toxicity score, and risk score is shown in Supplemental Table 1. An important feature of this analysis is to provide KC and HATs quartile ranking for 119 chemicals which lack an IARC risk assessment as human carcinogens.

The fraction of exposure occurrence to a given compound varies greatly between ZIP codes. Two patterns were observed, some ZIP codes reported many chemicals with 100% fraction of occurrence, whereas several ZIP codes reported only one chemical but with 100% occurrence. ZIP code 08014 reported the most chemicals with 100% emissions (1,1,2-tricloroethane, 2-nitropropane, 2,4-dinitrotoluene, benzidine, chlordane, chloroethane, heptachlor, hexachloroethane, methoxychlor, nitrobenzene, permethrin, thiram). See Supplemental Table 2 for details of the most reported chemical emissions by ZIP codes.

**Fig. 4:**
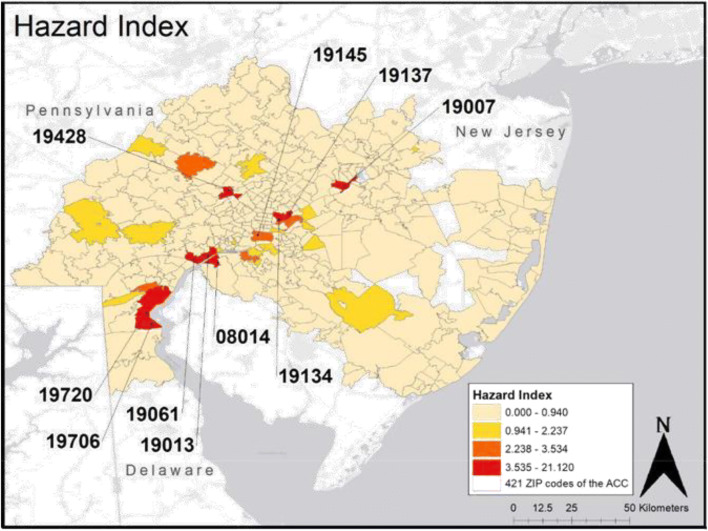
Hazard index for the 421 ZIP codes of the study area

The hazard index for the 421 ZIP codes in the study area ranged from a minimum of 0 (218 ZIP codes, 51.8%) to maximum of 21.12 (ZIP code 08014). A choropleth diagram of the hazard index mapped for each ZIP code is shown in Fig. 4. The median value among the 186 ZIP codes with a hazard index greater than 0 was 0.04. See the Supplemental Table 3 for details on the hazard index for all 421 ZIP codes.

Information about ZIP codes with the top 10 hazard indices, along with their emission summary, and population size is presented in Table 1. Of the top ten highest ZIP codes, 2,103,448 lbs of air emissions were released to Logan Township, NJ (08014), 806,738 lbs emitted to Conshohocken, PA (19428), 24,558,903 lbs emitted to Bristol, PA (19007), 8,295,962 lbs released to Port Richmond & Kensington, PA (19134), 15,945,469 lbs released to Bridesburg PA (19137), 19,652,501 lbs released to Marcus Hook, PA (19061), 1,071,511 lbs emitted to Chester (19013), 4,388,569 lbs released to New Castle, DE (19720), 8,842,667 lbs released to Delaware City, DE (19706), and 9,321,891 lbs released to PES Oil Refinery Region, PA (19145). Of these ten ZIP codes, 19706 is the only one not to border or intersect a major highway. See the Supplemental Table 4 for details on total air emissions of the 201 selected chemicals for all 421 ZIP codes. The list of 201 chemicals is in Supplemental Table 1.

**Table 1:** Top 10 ZIP codes in ACC study areas with the highest hazard index and high occurrence chemicals reported

ZIP code	Hazard index	Emissions (lbs)	County	Population size (2015 estimates)	Unique chemicals	Chemicals with >80% fraction of occurrence
Logan Township, NJ (08014)	21.12	2,103,448	Gloucester	504	66	1,1,2-Trichloroethane, 2-nitropropane, 2,4-dinitrotoluene, benzidine, chlordane, chloroethane, heptachlor, hexachloroethane, malathion, methoxychlor, nitrobenzene, permethrin, thiram, hexachlorobenzene, benzyl chloride, pyridine
Conshohocken, PA (19428)	6.44	806,738	Montgomery	16,580	38	Chlorothalonil, phenanthrene, anthracene
Bristol, PA (19007)	5.44	24,558,903	Bucks	21,125	29	Methyl acrylate, acrylamide, ethyl acrylate, acrylonitrile, methyl methacrylate
Port Richmond and Kensington, PA (19134)	5.07	8,295,962	Philadelphia	60,675	19	Phosphorus
Bridesburg, PA (19137)	4.98	15,945,469	Philadelphia	8,638	35	Benzoyl peroxide, chloromethane, phenol
Marcus Hook, PA (19061)	4.80	19,652,501	Delaware	19,997	38	All chemicals under 80% fraction of occurrence
Chester, PA (19013)	4.44	1,071,511	Delaware	35,130	19	Chloroform
New Castle, DE (19720)	4.18	4,388,569	New Castle	59,250	37	1,2-Dichlorobenzene, chlorobenzene, 1,2,4-Trichlorobenzene 1,3-Dichlorobenzene, 1,4-dichlorobenzene, epichlorohydrin
Delaware City, DE (19706)	4.04	8,842,667	New Castle	1,822	37	Carbon disulfide
PES Oil Refinery Region , PA (19145)	3.06	9,321,891	Philadelphia	47,261	29	o-Xylene

## Discussion

We investigated hazardous air exposure (exposomics) from anthropogenic sources in ZIP codes of a major US metropolitan area using EPA’s Toxic Release Inventory and NASA satellite data. Our results showed that there were varying exposures across the 421 ZIP codes under study; ZIP codes 19007 (Bristol, PA), 19310 (Atglen, PA), 19154 (Parkwood, PA), 19061 (Marcus Hook, PA), 19137 (Bridesburg, Philadelphia), 19426 (Collegeville, PA), 19365 (Parkesburg, PA), 19134 (Kensington/Port Richmond, PA), and 19145 (PES Oil Refinery Region, PA), 19720 (New Castle, DE) cumulatively had the greatest mean exposure to the hazardous chemicals that were important to air pollution and lung cancer risk. It should be noted that the PES oil refinery is no longer operational following an explosion in June 2019 and thus exposures from that point source will be reduced in the future.

Results from our MMCDA show that the ZIP codes with the highest hazard index include 08014 (Logan Township, NJ), 19428 (Conshohocken, PA), 19007 (Bristol, PA), 19134 (Port Richmond and Kensington, PA), 19137 (Bridesburg, PA), 19061 (Marcus Hook, PA), 19013 (Chester, PA), 19720 (New Castle, DE), 19706 (Delaware City, DE), and 19145 (PES Oil Refinery Region, PA). Some of these ZIP codes may not have the highest volume of emissions but contained proportionally high occurrence of a more toxic chemical or could be due to emissions from a large variety of chemicals that are toxic.

These ZIP codes tended to be in proximity to major highways which are important contributors to traffic-related air pollution in metropolitan areas. The predominate major highway in these high-risk ZIP codes is Interstate 95 (I-95) which covers approximately 1917 miles from Florida to Maine. In 2012, the U.S. Department of Transportation (DOT) reported the average daily traffic of the entire corridor at 72,000 with maximum levels reaching 300,000 and the average daily truck traffic at 10,000 with maximum levels extending over 31,000 (U.S. Department of Transportation, 2012).

Overall, the number of TRI facilities and their emissions has decreased from 1987 to 2017. This is encouraging news because facilities are either more environmentally conscious, or regulations have become more stringent. However, EPA TRI is not a complete picture of all potentially harmful emissions and comes with limitations. For example, the reporting of trade secret chemicals was not required before 2016 and 2017, adding uncertainty. The lack of information about these secret chemicals makes assessing their risk to environmental health difficult. Not all air-emitting industries are required to report chemical emissions to TRI, and not all chemicals are easily detectable. Reporting is conducted by the facility itself and not monitored directly by the EPA. Several significant industries in our study region are known to emit high levels of VOCs, NOx, and SOx but carry permits which allow them to not report to the TRI. The NASA satellite–derived NO_2_ and PM_2.5_ layers were only available as a shorter timeframe than the TRI information at the time of this study and limited the cumulative exposure outcome. The incorporation of other exposome data sources for this time period beyond the TRI data such as EJ screen (EPA 2018) would improve the hazard index.

The focus of this study is only on anthropogenic air pollution and lung cancer. Our analyses showed that Group 1 IARC chemicals made up 11.5% of all TRI air emissions, and VOCs consisted most of the reported emissions comprising 82.3%. Particularly hazardous VOCs (e.g., benzene, formaldehyde, butadiene, and acetaldehyde) were emitted in this study area, while certain troublesome PAHs (benzo[*a*]pyrene) or diesel exhaust (nitroarenes) were not. We were surprised to find that the exposure and therefore hazard indices are weighted much more in favor of VOCs than particulates such as PAH and nitroarenes. Future research could benefit from calculating evaporation rates by using vapor pressure for the volatile compounds. A significant number of PAHs were simply reported to the TRI as “polycyclic aromatic compounds,” with their speciation unknown. Although EPA air monitors capture concentrations on PM_2.5_, PM_10_, and NO_2_; hazardous air pollutants (HAPS); and volatile organic compounds (VOCs), NO/NO_x_/NO_y_, these data were very sparse for the study region and were excluded from the current analysis. Non-anthropogenic sources such as naturally occurring radon which can affect the incidence of lung cancer, or difficult-to-capture anthropogenic sources such as traffic and airport emissions, illegal emissions, and household activities can also contribute to pollution and thus add to the complexity to capturing the exposome for the study area.

The hazard index generated by the MMCDA framework provided further insight into this region’s exposure to lung carcinogens. An important feature of the MMCDA is the calculation of the chemical toxicity score which use KC’s and HATs to assess the carcinogenicity of 119 unknowns using citation searches from PubChem. This led to a risk assessment of these chemicals as carcinogens when none was available before. By weighing the frequencies of chemical occurrence by its propensity to cause cancer and environmental persistence, different ZIP codes came to our attention. In particular, the hazard index for ZIP code 08014 (Logan Township, NJ) was flagged as nearly threefold higher than the second highest scoring ZIP code 19428 (Conshohocken, PA). The annual age-adjusted lung cancer incidence rates for Gloucester county, which contains ZIP code 08014, consistently ranks in the top 2 or 3 highest rates in New Jersey. From years 2013 to 2017 for example, Gloucester 5-year lung cancer incidence rates were 74.6 (70.4, 78.9) per 100,000 compared to 55.3 (54.7, 56.0) for the state. Knowing that the hazard index for ZIP code 08014 is so high indicates a need to further investigate the surrounding area and assess the community’s health. Engaging smokers or other high-risk individuals in these elevated exposure areas to seek preventative care would be beneficial. The MMCDA developed for this study provides a novel tool in assessing a chemical’s carcinogenicity in a list of chemicals which considers both chemical toxicity, persistence, and occurrence. In particular, the proposed toxicity score captures the key characteristics of chemical carcinogens that has not been done before.

The urban areas found within the study region and the TRI facilities residing within are not unique compared to other US urban regions. Tobacco smoking and human proximity to lung cancer–causing emissions is an unfortunate human condition found across the nation and globe. If association between toxic environmental exposures and lung cancer holds true, then the prescription of a hazard index or analyses similar to what we performed may improve the efficacy of LCS. This approach could be used to identify high-risk areas where the effectives of screening could be assessed. By identifying smokers and never smokers who have lived in high-risk areas of exposure for extended periods of time we can sub-stratify at risk populations for participation in LCS trials to determine if there is an increase in lung cancer detection. The use of this MMCDA tool to develop hazard indices could be used in intervention trials to persuade smokers to participate in smoking cessation programs because of their higher risk. The hazard indices could also be used in lung cancer incidence surveillance programs to inform public health officials and decision makers to implement exposure reduction programs.

This study only examined toxic air exposures within the 12 counties of a metropolitan area. Air-polluting sources located near the study region, but not captured in this study, could be a significant source for future study. Meanwhile, the cumulative exposures created from publicly available EPA and NASA satellite data sources could be expanded to incorporate more years, additional layers (from EJ screen), or larger geographic areas of study. The methodology of this work could be used to determine risk of chemical exposures associated with other types of cancer to identify populations at risk.

## Supplementary information

Supplemental Table 1. 201 selected chemicals evaluated in the MMCDA with their computed KC, HAT and rescaled risk scores. (DOCX 79 kb)

Supplemental Table 2. ZIP codes in the study area with high fraction of chemical occurrence. (DOCX 14 kb)

Supplemental Table 3. Hazard Index for the ZIP codes in the study area. (DOCX 35 kb)

Supplemental Table 4. Total air emissions from 201 selected chemicals for ZIP codes in the study area. (DOCX 35 kb)

## Data Availability

The datasets used and analyzed during the current study are available from the corresponding author on reasonable request.

## Abbreviations

ALA: American Lung Association
HATs: “Human,” “animal,” “tumor”
ArcGIS: Geospatial mapping software
CDC: Centers for Disease Control and Prevention
DPM: Diesel particulate matter
KCs: Key characteristics
LCS: Lung cancer screening
MCDA: Multiple-criteria decision analysis
MMCDA: Multi-step multi-criteria decision analysis
NASA: National Aeronautics and Space Administration
NIH PubChem: National Institute of Health A database of chemical molecules and their activities
NO_2_: Nitrogen dioxide
PAHs: Polycyclic aromatic hydrocarbons
PES Oil refinery: Philadelphia Energy Solutions Oil Refinery
PM_2.5_: Particulate matter that has a diameter of less than 2.5 μm
US-EPA: United States-Environmental Protection Agency
TRI: Toxic Release Inventory
VOCs: Volatile organic compounds
WHO IARC: World Health Organization International Agency for Research on Cancer
ZIP codes: Zone Improvement Plan codes. Postal code used by the United States Postal Service
