# Supplementary material for: Environmental exposomics and lung cancer risk assessment in the Philadelphia metropolitan area using ZIP code–level hazard indices
Source: Environ Sci Pollut Res Int. Author manuscript; Available in PMC 2021 Jul 2. (PMC8238722; doi:10.1007/s11356-021-12884-z)
Supplement: Supplemental Table 2 [file NIHMS1676090-supplement-Supplemental_Table_2.docx]

**Supplemental Table 2.** ZIP codes in the study area with high fraction of chemical occurrence.

| **ZIP code** | **100% fraction of occurrence** | **<100% to 95% fraction of occurrence** | **<95% to 90% fraction of occurrence** |
| --- | --- | --- | --- |
| **08014** | 1,1,2-tricloroethane, 2-nitropropane, 2,4-dinitrotoluene, benzidine, chlordane, chloroethane, heptachlor, hexachloroethane, methoxychlor, nitrobenzene, permethrin, thiram, | hexachlorobenzene, malathion | benzyl chloride |
| **19720** |  | 1,2-dichlorobenzene, chlorobenzene, 1,2,4-trichlorobenzene, 1,3-dichlorobenzene, | 1,4-dichlorobenzene |
| **08011** | 4,4'-methylenedianiline, chloromethyl methyl ether |  |  |
| **19007** | methyl acrylate, acrylamide |  | ethyl acrylate, acrylonitrile |
| **19428** | chlorothalonil |  | phenanthrene |
| **19137** | benzoyl peroxide, chloromethane, |  | phenol |
| **19116** | bromomethane |  |  |
| **19013** | melamine |  | butyl benzyl phthalate |
| **19706** | carbon disulfide |  |  |
| **19148** | chlorendic acid, tetrabromobisphenol-a |  |  |
| **08052** | diglycidyl resorcinol ether |  |  |
| **08066** | hydrazine |  |  |
| **08037** |  |  | molybdenum trioxide |
| **08077** | n-methylolacrylamide |  |  |
| **19002** | o-toluidine |  |  |
| **08103** | polychlorinated biphenyls |  |  |
| **08561** | trifluralin |  |  |
| **08086** |  |  | vinylidene chloride |
