# Supplementary material for: Environmental exposomics and lung cancer risk assessment in the Philadelphia metropolitan area using ZIP code–level hazard indices
Source: Environ Sci Pollut Res Int. Author manuscript; Available in PMC 2021 Jul 2. (PMC8238722; doi:10.1007/s11356-021-12884-z)
Supplement: Supplemental Table 1 [file NIHMS1676090-supplement-Supplemental_Table_1.docx]

**Supplemental Table 1.** 201 selected chemicals evaluated in the MMCDA with their computed KC, HAT and rescaled risk scores

| Index | Chemical | Articles | IARC  Group | PAH | Component of Diesel Exhaust | VOC | Known Lung Carcinogen | KCs Score | HATs Score | Air Emissions | Risk Score  (Toxicity + Persistence Scores) |
| --- | --- | --- | --- | --- | --- | --- | --- | --- | --- | --- | --- |
| 1 | 1,1,1-Trichloroethane | 16 | 3 | No | No | No | No | 3 | 3 | 16123181 | 1.428571 |
| 2 | 1,1,2,2-Tetrachloroethane | 6 | No | No | No | Yes | No | 2 | 2 | 0 | 0.142857 |
| 3 | 1,1,2-Trichloro-1,2,2-trifluoroethane | 0 | No | No | No | Yes | No | 1 | 1 | 0 | 0 |
| 4 | 1,1,2-Trichloroethane | 6 | 3 | No | No | Yes | No | 2 | 2 | 18 | 0.285714 |
| 5 | 1,1-Dichloroethane | 4 | No | No | No | Yes | No | 2 | 2 | 0 | 0.142857 |
| 6 | 1,1-Dichloroethylene | 42 | No | No | No | Yes | No | 3 | 2 | 0 | 0.285714 |
| 7 | 1,2,3-Trimethylbenzene | 5 | No | No | No | Yes | No | 1 | 2 | 0 | 0.142857 |
| 8 | 1,2,4-Trichlorobenzene | 22 | No | No | No | Yes | No | 2 | 1 | 192419 | 0.142857 |
| 9 | 1,2,4-Trimethylbenzene | 4 | No | No | No | Yes | No | 1 | 2 | 1868996 | 0.142857 |
| 10 | 1,2-Dichlorobenzene | 31 | No | No | No | Yes | No | 3 | 2 | 365521 | 0.285714 |
| 11 | 1,2-Dichloroethane | 53 | 2B | No | No | No | No | 3 | 3 | 1095992 | 1.571429 |
| 12 | 1,2-Dichloropropane | 9 | 1 | No | No | Yes | No | 2 | 1 | 1649114.8 | 0.714286 |
| 13 | 1,3,5-Trimethylbenzene | 10 | No | No | No | Yes | No | 2 | 1 | 0 | 0.142857 |
| 14 | 1,3-Dichlorobenzene | 8 | No | No | No | Yes | No | 1 | 2 | 72402 | 0.142857 |
| 15 | 1,3-Diethylbenzene | 0 | No | No | No | Yes | No | 1 | 1 | 0 | 0 |
| 16 | 1,3-Dinitropyrene | 15 | No | No | Yes | No | No | 3 | 2 | 0 | 1.285714 |
| 17 | 1,4-Dichlorobenzene | 41 | No | No | No | Yes | No | 3 | 3 | 660055 | 0.285714 |
| 18 | 1,4-Dioxane | 70 | 2B | No | No | Yes | No | 1 | 2 | 114385.63 | 0.428571 |
| 19 | 1,6-Dinitropyrene | 23 | No | No | Yes | No | No | 3 | 3 | 0 | 1.285714 |
| 20 | 1-8 Dinitropyrene | 26 | No | No | Yes | No | No | 3 | 2 | 0 | 1.285714 |
| 21 | 1-Bromopropane | 12 | 2B | No | No | No | No | 2 | 1 | 302192 | 1.428571 |
| 22 | 1-Butene | 20 | No | No | No | Yes | No | 3 | 2 | 0 | 0.285714 |
| 23 | 1-Nitropyrene | 61 | No | No | Yes | No | No | 4 | 3 | 0 | 1.428571 |
| 24 | 1-Pentene | 1 | No | No | No | Yes | No | 1 | 1 | 0 | 0 |
| 25 | 2,2,4-Trimethylpentane | 4 | No | No | No | Yes | No | 2 | 2 | 0 | 0.142857 |
| 26 | 2,2-Dimethylbutane | 0 | No | No | No | Yes | No | 1 | 1 | 0 | 0 |
| 27 | 2,3,4-Trimethylpentane | 0 | No | No | No | Yes | No | 1 | 1 | 0 | 0 |
| 28 | 2,3-Dimethylbutane | 0 | No | No | No | Yes | No | 1 | 1 | 0 | 0 |
| 29 | 2,3-Dimethylpentane | 0 | No | No | No | Yes | No | 1 | 1 | 0 | 0 |
| 30 | 2,4-Dimethylpentane | 0 | No | No | No | Yes | No | 1 | 1 | 0 | 0 |
| 31 | 2,4-Dinitrotoluene | 7 | 2B | No | No | No | No | 1 | 1 | 21 | 1.285714 |
| 32 | 2-Butene | 15 | No | No | No | Yes | No | 3 | 3 | 0 | 0.285714 |
| 33 | 2-Chlorotoluene | 4 | No | No | No | Yes | No | 1 | 1 | 0 | 0 |
| 34 | 2-Methylheptane | 12 | No | No | No | Yes | No | 2 | 2 | 0 | 0.142857 |
| 35 | 2-Methylhexane | 0 | No | No | No | Yes | No | 1 | 1 | 0 | 0 |
| 36 | 2-Methylpentane | 2 | No | No | No | Yes | No | 1 | 1 | 0 | 0 |
| 37 | 2-Nitrofluorene | 32 | No | No | Yes | No | No | 3 | 3 | 0 | 1.285714 |
| 38 | 2-Nitropropane | 23 | 2B | No | No | No | No | 3 | 1 | 7 | 1.571429 |
| 39 | 3,7-Dinitrofluoranthene | 2 | No | No | Yes | No | No | 1 | 1 | 0 | 1 |
| 40 | 3,9-Dinitrofluoranthene | 4 | No | No | Yes | No | No | 1 | 1 | 0 | 1 |
| 41 | 3-Chloropropene | 3 | No | No | No | Yes | No | 1 | 1 | 0 | 0 |
| 42 | 3-Methylheptane | 6 | No | No | No | Yes | No | 1 | 2 | 0 | 0.142857 |
| 43 | 3-Methylhexane | 0 | No | No | No | Yes | No | 1 | 1 | 0 | 0 |
| 44 | 3-Methylpentane | 0 | No | No | No | Yes | No | 1 | 1 | 0 | 0 |
| 45 | 3-Nitrobenzanthrone | 34 | No | No | Yes | No | No | 3 | 3 | 0 | 1.285714 |
| 46 | 4,4'-Methylenedianiline | 10 | 2B | No | No | No | No | 2 | 1 | 176 | 1.428571 |
| 47 | 4-Nitropyrene | 2 | No | No | Yes | No | No | 2 | 1 | 0 | 1.142857 |
| 48 | 6-Nitrochrysene | 17 | No | No | Yes | No | No | 3 | 3 | 0 | 1.285714 |
| 49 | Acenaphthene | 21 | No | Yes | No | No | No | 2 | 2 | 0 | 1.142857 |
| 50 | Acenaphthylene | 12 | No | Yes | No | No | No | 2 | 2 | 0 | 1.142857 |
| 51 | Acetaldehyde | 601 | 2B | No | Yes | Yes | No | 4 | 4 | 227423.8 | 0.714286 |
| 52 | Acetone | 587 | No | No | No | Yes | No | 4 | 4 | 6951077 | 0.428571 |
| 53 | Acetonitrile | 380 | No | No | No | Yes | No | 4 | 4 | 144746.75 | 0.428571 |
| 54 | Acetylene | 61 | No | No | No | Yes | No | 4 | 4 | 0 | 0.428571 |
| 55 | Acrolein | 809 | No | No | No | Yes | No | 4 | 4 | 0 | 0.428571 |
| 56 | Acrylamide | 845 | 2A | No | No | No | No | 2 | 2 | 7043 | 1.571429 |
| 57 | Acrylic Acid | 97 | 3 | No | No | No | No | 3 | 3 | 78941.17 | 1.428571 |
| 58 | Acrylonitrile | 120 | 2B | No | No | Yes | No | 4 | 4 | 18001.3 | 0.714286 |
| 59 | Aluminum | 216 | No | No | No | No | Yes | 4 | 3 | 1790537.3 | 1.428571 |
| 60 | Aniline | 763 | 3 | No | No | No | No | 1 | 1 | 6749 | 1.142857 |
| 61 | Anthracene | 1,004 | 3 | Yes | No | No | No | 4 | 4 | 3716.24 | 1.571429 |
| 62 | Arsenic | 190 | No | No | Yes | No | Yes | 4 | 3 | 18105.499 | 1.428571 |
| 63 | Asbestos | 91 | 1 | No | No | No | Yes | 3 | 3 | 3274 | 1.857143 |
| 64 | Benz(A)anthracene | 94 | No | Yes | No | No | No | 3 | 3 | 0 | 1.285714 |
| 65 | Benzene | 2,303 | 1 | No | Yes | Yes | Yes | 4 | 4 | 5237997.4 | 1 |
| 66 | Benzidine | 79 | 1 | No | No | No | No | 3 | 3 | 3 | 1.857143 |
| 67 | Benzo(A)pyrene | 1,843 | No | Yes | No | No | No | 4 | 4 | 0 | 1.428571 |
| 68 | Benzo(B)fluoranthene | 49 | No | Yes | No | No | No | 3 | 3 | 0 | 1.285714 |
| 69 | Benzo(k)fluoranthene | 39 | No | Yes | No | No | No | 3 | 3 | 0 | 1.285714 |
| 70 | Benzo[G,H,I]Perylene | 16 | No | Yes | No | No | No | 2 | 2 | 477.2249 | 1.142857 |
| 71 | Benzoyl Chloride | 4 | No | No | No | No | Yes | 1 | 2 | 0 | 1.142857 |
| 72 | Benzoyl Peroxide | 90 | 3 | No | No | No | No | 3 | 3 | 1 | 1.428571 |
| 73 | Benzyl Chloride | 16 | No | No | No | Yes | No | 2 | 1 | 83884 | 0.142857 |
| 74 | Beryllium | 47 | 1 | No | No | No | Yes | 3 | 3 | 1277 | 1.857143 |
| 75 | Biphenyl | 1,624 | No | No | No | No | No | 4 | 4 | 46497.79 | 1.428571 |
| 76 | Bis (Chloromethyl) Ether | 1 | 1 | No | No | No | Yes | 1 | 2 | 0 | 1.714286 |
| 77 | Bromochloromethane | 5 | No | No | No | Yes | No | 1 | 1 | 0 | 0 |
| 78 | Bromodichloromethane | 23 | No | No | No | Yes | No | 2 | 3 | 0 | 0.285714 |
| 79 | Bromoform | 12 | No | No | No | Yes | No | 2 | 3 | 0 | 0.285714 |
| 80 | Bromomethane | 1 | No | No | No | Yes | No | 1 | 1 | 12000 | 0 |
| 81 | Butadiene | 240 | No | No | Yes | No | No | 4 | 4 | 252759.4 | 1.428571 |
| 82 | Butyl Benzyl Phthalate | 27 | 3 | No | No | No | No | 3 | 3 | 480940 | 1.428571 |
| 83 | Cadmium | 1204 | 1 | No | No | No | Yes | 4 | 4 | 19999 | 2 |
| 84 | Carbon Disulfide | 110 | No | No | No | Yes | No | 3 | 3 | 211844 | 0.285714 |
| 85 | Carbon Tetrachloride | 1,573 | 2B | No | No | Yes | No | 4 | 4 | 1066738 | 0.714286 |
| 86 | Chlordane | 10 | 2B | No | No | No | No | 2 | 3 | 25.44 | 1.571429 |
| 87 | Chlorendic Acid | 3 | 2B | No | No | No | No | 1 | 1 | 237 | 1.285714 |
| 88 | Chlorobenzene | 85 | No | No | No | Yes | No | 3 | 3 | 897231 | 0.285714 |
| 89 | Chlorodifluoromethane | 0 | 3 | No | No | No | No | 3 | 3 | 4403218.2 | 1.428571 |
| 90 | Chloroethane | 11 | 3 | No | No | Yes | No | 2 | 2 | 1395207 | 0.285714 |
| 91 | Chloroform | 573 | 2B | No | No | Yes | No | 4 | 4 | 482924 | 0.714286 |
| 92 | Chloromethane | 3 | No | No | No | Yes | No | 2 | 2 | 414633 | 0.142857 |
| 93 | Chloromethyl Methyl Ether | 3 | 1 | No | No | No | No | 2 | 2 | 30035 | 1.714286 |
| 94 | Chloroprene | 12 | No | No | No | Yes | No | 2 | 2 | 0 | 0.142857 |
| 95 | Chlorothalonil | 25 | 2B | No | No | No | No | 2 | 2 | 269 | 1.428571 |
| 96 | Chromium Compounds | 448 | 1 | No | Yes | No | Yes | 4 | 4 | 579766.58 | 2 |
| 97 | Chrysene | 66 | No | Yes | No | No | No | 3 | 3 | 0 | 1.285714 |
| 98 | cis-1 3-dichloropropene | 4 | No | No | No | Yes | No | 2 | 1 | 0 | 0.142857 |
| 99 | cis-1,2-Dicloroethene | 14 | No | No | No | Yes | No | 1 | 1 | 0 | 0 |
| 100 | cis-2-Butene | 9 | No | No | No | Yes | No | 2 | 2 | 0 | 0.142857 |
| 101 | cis-2-Pentene | 1 | No | No | No | Yes | No | 1 | 1 | 0 | 0 |
| 102 | Cumene | 37 | 2B | No | No | No | No | 3 | 2 | 13520922 | 1.571429 |
| 103 | Cyclohexane | 80 | No | No | No | Yes | No | 4 | 4 | 2403528.6 | 0.428571 |
| 104 | Cyclopentane | 28 | No | No | No | Yes | No | 3 | 3 | 0 | 0.285714 |
| 105 | Decabromodiphenyl Oxide | 24 | 3 | No | No | No | No | 2 | 3 | 5949.0372 | 1.428571 |
| 106 | Dibenz(a,h)anthracene | 51 | No | Yes | No | No | No | 3 | 3 | 0 | 1.285714 |
| 107 | Dibromochloromethane | 7 | No | No | No | Yes | No | 1 | 2 | 0 | 0.142857 |
| 108 | Dichlorodifluoromethane | 1 | No | No | No | Yes | No | 1 | 2 | 1155152 | 0.142857 |
| 109 | Dichloromethane | 437 | No | No | No | Yes | No | 1 | 1 | 6320040.2 | 0 |
| 110 | Dichlorotetrafluoroethane | 0 | No | No | No | Yes | No | 1 | 1 | 0 | 0 |
| 111 | Diethanolamine | 43 | 2B | No | No | No | No | 3 | 3 | 121566.64 | 1.571429 |
| 112 | Diethyl Sulfate | 27 | 2A | No | No | No | No | 3 | 2 | 7800.2 | 1.714286 |
| 113 | Diglycidyl Resorcinol Ether | 2 | 2B | No | No | No | No | 1 | 1 | 580.01 | 1.285714 |
| 114 | Dioxin | 745 | 1 | No | No | No | No | 4 | 4 | 0.428128 | 2 |
| 115 | Epichlorohydrin | 28 | 2A | No | No | No | No | 3 | 3 | 49975.64 | 1.714286 |
| 116 | Ethane | 435 | No | No | No | Yes | No | 4 | 4 | 0 | 0.428571 |
| 117 | Ethyl Acetate | 823 | No | No | No | Yes | No | 4 | 4 | 0 | 0.428571 |
| 118 | Ethyl Acrylate | 27 | 2B | No | No | Yes | No | 3 | 3 | 378747.4 | 0.571429 |
| 119 | Ethylbenzene | 199 | 2B | No | No | Yes | No | 4 | 4 | 2561816.1 | 0.714286 |
| 120 | Ethylene | 2,191 | 3 | No | No | Yes | No | 4 | 4 | 2198232.9 | 0.571429 |
| 121 | Ethylene dibromide | 44 | No | No | No | Yes | No | 3 | 2 | 0 | 0.285714 |
| 122 | Ethylene dichloride | 4 | No | No | No | Yes | No | 2 | 2 | 0 | 0.142857 |
| 123 | Ethylene Oxide | 290 | 1 | No | No | No | No | 4 | 4 | 1281555 | 2 |
| 124 | Fluoranthene | 114 | No | Yes | No | No | No | 3 | 3 | 0 | 1.285714 |
| 125 | Fluorene | 81 | No | Yes | No | No | No | 3 | 3 | 0 | 1.285714 |
| 126 | Formaldehyde | 1,538 | 1 | No | Yes | Yes | No | 4 | 4 | 1090539.2 | 1 |
| 127 | Heptachlor | 23 | 2B | No | No | No | No | 2 | 3 | 11.6 | 1.571429 |
| 128 | Hexachlorobenzene | 71 | 2B | No | No | No | No | 3 | 4 | 123.71138 | 1.714286 |
| 129 | Hexachlorobutadiene | 10 | No | No | No | Yes | No | 2 | 2 | 0 | 0.142857 |
| 130 | Hexachloroethane | 7 | 2B | No | No | No | N | 2 | 1 | 22 | 1.428571 |
| 131 | Hydrazine | 225 | 2A | No | No | No | Yes | 4 | 4 | 27 | 1.857143 |
| 132 | Hydroquinone | 451 | 3 | No | No | No | No | 4 | 4 | 565 | 1.571429 |
| 133 | indeno(1,2,3-cd)pyrene | 22 | No | Yes | No | No | No | 2 | 2 | 0 | 1.142857 |
| 134 | Isobutane | 6 | No | No | No | Yes | No | 2 | 2 | 0 | 0.142857 |
| 135 | Isopentane | 1 | No | No | No | Yes | No | 1 | 3 | 0 | 0.285714 |
| 136 | Isoprene | 67 | No | No | No | Yes | No | 4 | 4 | 0 | 0.428571 |
| 137 | Isopropyl Alcohol | 1,710 | 3 | No | No | No | No | 4 | 4 | 51195 | 1.571429 |
| 138 | Isopropylbenzene | 40 | No | No | No | Yes | No | 3 | 2 | 0 | 0.285714 |
| 139 | Lead | 421 | 2B | No | No | No | No | 4 | 4 | 383115.33 | 1.714286 |
| 140 | Malathion | 124 | 2A | No | No | No | No | 3 | 4 | 2 | 1.857143 |
| 141 | Melamine | 31 | 2B | No | No | No | No | 3 | 3 | 6 | 1.571429 |
| 142 | Methoxychlor | 86 | 3 | No | No | No | No | 3 | 4 | 2.2 | 1.571429 |
| 143 | Methyl Acrylate | 10 | 2B | No | No | No | No | 2 | 2 | 12953.4 | 1.428571 |
| 144 | Methyl Chloroform | 31 | No | No | No | Yes | No | 3 | 3 | 0 | 0.285714 |
| 145 | Methyl Ethyl Ketone | 13 | No | No | No | Yes | No | 3 | 3 | 21221463 | 0.285714 |
| 146 | Methyl Isobutyl Ketone | 3 | 2B | No | No | Yes | No | 2 | 3 | 7695245.9 | 0.571429 |
| 147 | Methyl Methacrylate | 67 | 3 | No | No | Yes | No | 4 | 4 | 2608409.2 | 0.571429 |
| 148 | Methyl Tert-Butyl Ether | 30 | 3 | No | No | Yes | No | 3 | 4 | 3321101.4 | 0.571429 |
| 149 | Methylcyclohexane | 4 | No | No | No | Yes | No | 1 | 1 | 0 | 0 |
| 150 | Methylcyclopentane | 1 | No | No | No | Yes | No | 1 | 1 | 0 | 0 |
| 151 | Molybdenum Trioxide | 0 | 2B | No | No | No | No | 1 | 1 | 143386.14 | 1.285714 |
| 152 | n,n-Dimethylformamide | 220 | 2A | No | No | No | No | 4 | 4 | 1121137.9 | 1.857143 |
| 153 | Naphthalene | 554 | 2B | Yes | No | No | No | 4 | 4 | 888940.07 | 1.714286 |
| 154 | n-Butane | 2 | No | No | No | Yes | No | 2 | 1 | 0 | 0.142857 |
| 155 | n-Decane | 2 | No | No | No | Yes | No | 2 | 2 | 0 | 0.142857 |
| 156 | n-Heptane | 1 | No | No | No | Yes | No | 2 | 3 | 0 | 0.285714 |
| 157 | n-Hexane | 159 | No | No | No | Yes | No | 4 | 4 | 3894522.5 | 0.428571 |
| 158 | Nickel Compounds | 632 | 1 | No | No | No | Yes | 4 | 4 | 658618.58 | 2 |
| 159 | Nitrobenzene | 152 | 2B | No | No | No | No | 3 | 3 | 59 | 1.571429 |
| 160 | n-Methylolacrylamide | 0 | 3 | No | No | No | No | 1 | 1 | 1016 | 1.142857 |
| 161 | n-Nonane | 0 | No | No | No | Yes | No | 2 | 1 | 0 | 0.142857 |
| 162 | n-Octane | 3 | No | No | No | Yes | No | 2 | 2 | 0 | 0.142857 |
| 163 | n-Pentane | 6 | No | No | Yes | Yes | No | 2 | 3 | 0 | 0.285714 |
| 164 | n-Propylbenzene | 18 | No | No | No | Yes | No | 2 | 2 | 0 | 0.142857 |
| 165 | n-Undecane | 10 | No | No | No | Yes | No | 2 | 2 | 0 | 0.142857 |
| 166 | o-ethyltoluene | 1 | No | No | No | Yes | No | 1 | 1 | 0 | 0 |
| 167 | o-toluidine | 18 | 1 | No | No | No | No | 1 | 1 | 3275 | 1.571429 |
| 168 | o-Xylene | 1,482 | No | No | No | Yes | No | 4 | 4 | 178775.93 | 0.428571 |
| 169 | p-Diethylbenzene | 1 | No | No | No | Yes | No | 1 | 1 | 0 | 0 |
| 170 | Permethrin | 49 | 3 | No | No | No | No | 3 | 3 | 1 | 1.428571 |
| 171 | p-ethyltoluene | 0 | No | No | No | Yes | No | 1 | 1 | 0 | 0 |
| 172 | Phenanthrene | 287 | 3 | Yes | No | No | No | 4 | 3 | 10997.31 | 1.571429 |
| 173 | Phenol | 2729 | 3 | No | No | No | No | 4 | 4 | 2735347.3 | 1.571429 |
| 174 | Phosphorus | 1,253 | 1 | No | No | No | No | 4 | 4 | 15030 | 2 |
| 175 | Polychlorinated Biphenyls | 0 | No | No | No | No | No | 1 | 1 | 7.79282 | 1 |
| 176 | Polycyclic Aromatic Compounds | 0 | No | Yes | No | No | No | 1 | 1 | 61780.46 | 1 |
| 177 | Propane | 233 | No | No | No | Yes | No | 4 | 4 | 0 | 0.428571 |
| 178 | Propylene | 1,323 | 3 | No | No | Yes | No | 4 | 4 | 6059007.6 | 0.571429 |
| 179 | Propylene Oxide | 126 | 2B | No | No | No | No | 2 | 2 | 946610.34 | 1.428571 |
| 180 | Pyrene | 541 | No | Yes | No | No | No | 4 | 4 | 0 | 1.428571 |
| 181 | Pyridine | 1037 | 2B | No | No | No | No | 4 | 4 | 101 | 1.714286 |
| 182 | Quinoline | 391 | 2B | No | No | No | No | 4 | 4 | 192 | 1.714286 |
| 183 | Styrene | 338 | 2A | No | No | Yes | No | 4 | 4 | 4380334.1 | 0.857143 |
| 184 | Tert-amyl methyl ether | 4 | No | No | No | Yes | No | 1 | 2 | 0 | 0.142857 |
| 185 | Tert-Butyl Alcohol | 38 | No | No | No | Yes | No | 3 | 3 | 36066 | 0.285714 |
| 186 | Tert-Butyl-Ethyl-Ether | 2 | No | No | No | Yes | No | 2 | 3 | 0 | 0.285714 |
| 187 | Tetrabromobisphenol A | 24 | 2A | No | No | No | No | 2 | 3 | 1208 | 1.714286 |
| 188 | Tetrachloroethylene | 26 | No | No | No | Yes | No | 4 | 4 | 1122848.2 | 0.428571 |
| 189 | Thiram | 16 | 3 | No | No | No | No | 2 | 2 | 1 | 1.285714 |
| 190 | Toluene | 1,439 | 3 | No | No | Yes | No | 4 | 4 | 80409770 | 0.571429 |
| 191 | Trans-1,2-Dichloroethylene | 14 | No | No | No | Yes | No | 2 | 1 | 0 | 0.142857 |
| 192 | Trans-1,3-dichloropropene | 6 | No | No | No | Yes | No | 2 | 2 | 0 | 0.142857 |
| 193 | Trans-2-Pentene | 0 | No | No | No | Yes | No | 1 | 1 | 0 | 0 |
| 194 | Trichloroethylene | 153 | 1 | No | No | Yes | No | 4 | 4 | 14443923 | 1 |
| 195 | Trichlorofluoromethane | 1 | No | No | No | Yes | No | 1 | 1 | 466101 | 0 |
| 196 | Trifluralin | 8 | 3 | No | No | No | No | 1 | 2 | 1000 | 1.285714 |
| 197 | Vinyl Acetate | 39 | 2B | No | No | Yes | No | 3 | 4 | 3091508.6 | 0.714286 |
| 198 | Vinyl bromide | 9 | No | No | No | Yes | No | 2 | 1 | 0 | 0.142857 |
| 199 | Vinyl chloride | 181 | 1 | No | No | Yes | No | 4 | 3 | 5921140 | 1 |
| 200 | Vinylidene Chloride | 38 | 2B | No | No | No | No | 3 | 2 | 248 | 1.571429 |
| 201 | Xylene (Mixed Isomers) | 6 | No | No | No | Yes | No | 1 | 1 | 27758586 | 0 |
