# Supplementary material for: Environmental exposomics and lung cancer risk assessment in the Philadelphia metropolitan area using ZIP code–level hazard indices
Source: Environ Sci Pollut Res Int. Author manuscript; Available in PMC 2021 Jul 2. (PMC8238722; doi:10.1007/s11356-021-12884-z)
Supplement: Supplemental Table 3 [file NIHMS1676090-supplement-Supplemental_Table_3.docx]

**Supplemental Table 3.** Hazard Index for the ZIP codes in the study area.

| **ZIP code** | **Final Hazard Index** |  | **ZIP code** | **Final Hazard Index** |  | **ZIP code** | **Final Hazard Index** |  | **ZIP code** | **Final Hazard Index** |
| --- | --- | --- | --- | --- | --- | --- | --- | --- | --- | --- |
| 08014 | 21.11990406 |  | 08330 | 0.033482483 |  | 08080 | 0 |  | 19437 | 0 |
| 19428 | 6.43802646 |  | 18944 | 0.031719655 |  | 08095 | 0 |  | 19444 | 0 |
| 19007 | 5.440873959 |  | 19146 | 0.031614743 |  | 08350 | 0 |  | 19453 | 0 |
| 19134 | 5.071504101 |  | 08007 | 0.029196045 |  | 19102 | 0 |  | 19456 | 0 |
| 19137 | 4.980748371 |  | 19125 | 0.028860673 |  | 19103 | 0 |  | 19465 | 0 |
| 19061 | 4.800697525 |  | 18073 | 0.026219375 |  | 19104 | 0 |  | 08217 | 0 |
| 19013 | 4.440701174 |  | 19047 | 0.025557953 |  | 19109 | 0 |  | 08221 | 0 |
| 19720 | 4.183065872 |  | 19405 | 0.024295061 |  | 19113 | 0 |  | 08225 | 0 |
| 19706 | 4.04339711 |  | 19029 | 0.023717704 |  | 19119 | 0 |  | 19472 | 0 |
| 19145 | 3.055477165 |  | 18969 | 0.023074518 |  | 19122 | 0 |  | 19473 | 0 |
| 19148 | 3.038128257 |  | 19121 | 0.02250721 |  | 19126 | 0 |  | 19475 | 0 |
| 19804 | 2.75614774 |  | 19014 | 0.022292816 |  | 19128 | 0 |  | 19492 | 0 |
| 19426 | 2.691832708 |  | 19090 | 0.021084713 |  | 19138 | 0 |  | 08232 | 0 |
| 08011 | 2.646482921 |  | 19390 | 0.020454409 |  | 19139 | 0 |  | 08241 | 0 |
| 08066 | 2.604117353 |  | 19301 | 0.02016686 |  | 19141 | 0 |  | 08244 | 0 |
| 08110 | 2.55182705 |  | 19006 | 0.020104156 |  | 19147 | 0 |  | 08317 | 0 |
| 19002 | 2.186528574 |  | 19153 | 0.019986869 |  | 08097 | 0 |  | 08319 | 0 |
| 19022 | 2.033481325 |  | 19133 | 0.019389718 |  | 19149 | 0 |  | 08322 | 0 |
| 19320 | 1.705876576 |  | 19053 | 0.018518894 |  | 19150 | 0 |  | 08326 | 0 |
| 08086 | 1.650624917 |  | 19127 | 0.018113085 |  | 19151 | 0 |  | 08328 | 0 |
| 19713 | 1.62621993 |  | 08641 | 0.01772387 |  | 19152 | 0 |  | 19707 | 0 |
| 08052 | 1.567375538 |  | 19477 | 0.016984419 |  | 19803 | 0 |  | 19710 | 0 |
| 08093 | 1.561917406 |  | 08690 | 0.016607709 |  | 18902 | 0 |  | 19716 | 0 |
| 19382 | 1.494518552 |  | 19124 | 0.016377014 |  | 18912 | 0 |  | 19717 | 0 |
| 08003 | 1.430585987 |  | 19073 | 0.016278483 |  | 18913 | 0 |  | 19730 | 0 |
| 08077 | 1.371749294 |  | 19132 | 0.015413384 |  | 18917 | 0 |  | 19731 | 0 |
| 19032 | 1.332145889 |  | 19801 | 0.015323737 |  | 18920 | 0 |  | 19732 | 0 |
| 08561 | 1.29687109 |  | 19087 | 0.014378897 |  | 18923 | 0 |  | 19733 | 0 |
| 19464 | 1.266923725 |  | 19040 | 0.013673561 |  | 18925 | 0 |  | 19734 | 0 |
| 08037 | 1.202292738 |  | 08310 | 0.013021081 |  | 18929 | 0 |  | 19735 | 0 |
| 08103 | 1.197746542 |  | 18940 | 0.013021072 |  | 18930 | 0 |  | 19736 | 0 |
| 18936 | 1.077524725 |  | 19142 | 0.010238409 |  | 19805 | 0 |  | 19072 | 0 |
| 19809 | 0.882455249 |  | 08002 | 0.010015829 |  | 19807 | 0 |  | 19074 | 0 |
| 19380 | 0.864504191 |  | 18914 | 0.009840864 |  | 19810 | 0 |  | 19075 | 0 |
| 08102 | 0.855055375 |  | 19462 | 0.009232657 |  | 18932 | 0 |  | 19076 | 0 |
| 19442 | 0.853364687 |  | 19701 | 0.007340444 |  | 18935 | 0 |  | 19079 | 0 |
| 19703 | 0.845304862 |  | 19143 | 0.007147984 |  | 18938 | 0 |  | 19081 | 0 |
| 08036 | 0.780170852 |  | 08048 | 0.007045674 |  | 18942 | 0 |  | 19082 | 0 |
| 19358 | 0.762345537 |  | 08608 | 0.006940153 |  | 18947 | 0 |  | 19083 | 0 |
| 08016 | 0.734864142 |  | 08550 | 0.006780755 |  | 18950 | 0 |  | 19085 | 0 |
| 08638 | 0.718428496 |  | 18964 | 0.006696066 |  | 18954 | 0 |  | 19086 | 0 |
| 19030 | 0.594522616 |  | 19135 | 0.006522268 |  | 18955 | 0 |  | 19094 | 0 |
| 19067 | 0.50397339 |  | 08081 | 0.006054128 |  | 18962 | 0 |  | 19095 | 0 |
| 08009 | 0.482648613 |  | 19438 | 0.005993946 |  | 18972 | 0 |  | 19096 | 0 |
| 19446 | 0.480798164 |  | 19131 | 0.005723435 |  | 18977 | 0 |  | 08341 | 0 |
| 19348 | 0.441546103 |  | 19064 | 0.005697575 |  | 18980 | 0 |  | 08346 | 0 |
| 19154 | 0.412472791 |  | 19468 | 0.005140912 |  | 19001 | 0 |  | 08401 | 0 |
| 08619 | 0.383605965 |  | 08094 | 0.004798218 |  | 19003 | 0 |  | 08402 | 0 |
| 08028 | 0.365081217 |  | 18976 | 0.004270087 |  | 19009 | 0 |  | 08403 | 0 |
| 18041 | 0.330218812 |  | 19144 | 0.004142306 |  | 19012 | 0 |  | 08406 | 0 |
| 19057 | 0.329318513 |  | 19808 | 0.003895517 |  | 19017 | 0 |  | 08515 | 0 |
| 19130 | 0.322791482 |  | 19341 | 0.003742578 |  | 19025 | 0 |  | 08525 | 0 |
| 19310 | 0.3062102 |  | 08759 | 0.003051758 |  | 19026 | 0 |  | 08527 | 0 |
| 08027 | 0.305362349 |  | 19107 | 0.002662999 |  | 19027 | 0 |  | 08540 | 0 |
| 19046 | 0.273555218 |  | 08056 | 0.002247503 |  | 19031 | 0 |  | 08542 | 0 |
| 08224 | 0.248729152 |  | 19350 | 0.002054401 |  | 19033 | 0 |  | 08004 | 0 |
| 19702 | 0.210802411 |  | 18070 | 0.001747237 |  | 19035 | 0 |  | 08005 | 0 |
| 08611 | 0.202709941 |  | 08753 | 0.001720207 |  | 19041 | 0 |  | 08006 | 0 |
| 19454 | 0.19421638 |  | 19021 | 0.001631323 |  | 19043 | 0 |  | 08008 | 0 |
| 18966 | 0.188175939 |  | 08234 | 0.00159286 |  | 19054 | 0 |  | 08010 | 0 |
| 08057 | 0.179769915 |  | 08075 | 0.001442984 |  | 19055 | 0 |  | 08015 | 0 |
| 08344 | 0.1753749 |  | 19401 | 0.001432023 |  | 19056 | 0 |  | 08019 | 0 |
| 08215 | 0.174952096 |  | 19422 | 0.0014242 |  | 19060 | 0 |  | 08554 | 0 |
| 19355 | 0.172769584 |  | 08065 | 0.001262686 |  | 19063 | 0 |  | 08560 | 0 |
| 08030 | 0.172761951 |  | 08312 | 0.001207057 |  | 19066 | 0 |  | 08562 | 0 |
| 19403 | 0.165027546 |  | 08533 | 0.0012003 |  | 19070 | 0 |  | 08610 | 0 |
| 18974 | 0.139077582 |  | 08620 | 0.001122027 |  | 08059 | 0 |  | 08629 | 0 |
| 08085 | 0.137430883 |  | 08691 | 0.001116725 |  | 08061 | 0 |  | 08640 | 0 |
| 08105 | 0.130579342 |  | 19106 | 0.000959584 |  | 08062 | 0 |  | 18054 | 0 |
| 08104 | 0.123142827 |  | 19015 | 0.000945881 |  | 08063 | 0 |  | 08721 | 0 |
| 19112 | 0.117886105 |  | 19123 | 0.000923444 |  | 08064 | 0 |  | 08722 | 0 |
| 19709 | 0.11784404 |  | 19129 | 0.000837374 |  | 08068 | 0 |  | 08723 | 0 |
| 19140 | 0.117826621 |  | 19120 | 0.000806221 |  | 08074 | 0 |  | 08731 | 0 |
| 19474 | 0.114281146 |  | 19806 | 0.000687733 |  | 08078 | 0 |  | 08732 | 0 |
| 19044 | 0.106160202 |  | 08609 | 0.000554723 |  | 08083 | 0 |  | 08734 | 0 |
| 19344 | 0.091283885 |  | 19115 | 0.00055301 |  | 08084 | 0 |  | 08735 | 0 |
| 19460 | 0.090528485 |  | 19023 | 0.000516743 |  | 08087 | 0 |  | 08738 | 0 |
| 19335 | 0.086500783 |  | 08534 | 0.000495487 |  | 08089 | 0 |  | 08740 | 0 |
| 08096 | 0.086146912 |  | 19038 | 0.000398923 |  | 08090 | 0 |  | 18074 | 0 |
| 08518 | 0.084945593 |  | 18915 | 0.000375449 |  | 08091 | 0 |  | 18076 | 0 |
| 08648 | 0.081049485 |  | 19008 | 0.000305578 |  | 08092 | 0 |  | 18077 | 0 |
| 08071 | 0.080514186 |  | 08060 | 0.000291365 |  | 19312 | 0 |  | 18081 | 0 |
| 19406 | 0.080463002 |  | 19520 | 0.000246041 |  | 19316 | 0 |  | 08020 | 0 |
| 18960 | 0.076409373 |  | 08088 | 0.000129491 |  | 19317 | 0 |  | 08021 | 0 |
| 19050 | 0.075823988 |  | 08055 | 9.47E-05 |  | 19319 | 0 |  | 08022 | 0 |
| 19802 | 0.075503561 |  | 08733 | 8.43E-05 |  | 19333 | 0 |  | 08029 | 0 |
| 19078 | 0.074296314 |  | 19711 | 4.64E-05 |  | 19342 | 0 |  | 08033 | 0 |
| 18951 | 0.071978439 |  | 19004 | 3.76E-05 |  | 19343 | 0 |  | 08741 | 0 |
| 08505 | 0.070358398 |  | 08618 | 1.78E-05 |  | 19345 | 0 |  | 08742 | 0 |
| 19136 | 0.069624477 |  | 08511 | 1.23E-05 |  | 19352 | 0 |  | 08751 | 0 |
| 19116 | 0.066978112 |  | 08012 | 9.29E-06 |  | 19362 | 0 |  | 08752 | 0 |
| 19363 | 0.065881833 |  | 19111 | 7.11E-06 |  | 19365 | 0 |  | 08757 | 0 |
| 19020 | 0.063539781 |  | 19372 | 4.47E-06 |  | 19367 | 0 |  | 08758 | 0 |
| 08240 | 0.062601244 |  | 08755 | 6.17E-07 |  | 19373 | 0 |  | 08035 | 0 |
| 08034 | 0.060850022 |  | 08724 | 4.47E-07 |  | 19374 | 0 |  | 08039 | 0 |
| 19457 | 0.057498802 |  | 19330 | 4.47E-08 |  | 19375 | 0 |  | 08041 | 0 |
| 19440 | 0.054557529 |  | 19311 | 1.04E-08 |  | 19383 | 0 |  | 08042 | 0 |
| 19018 | 0.052635517 |  | 18970 | 0 |  | 19425 | 0 |  | 08043 | 0 |
| 19034 | 0.051416261 |  | 19010 | 0 |  | 08106 | 0 |  | 08045 | 0 |
| 08026 | 0.050919811 |  | 19036 | 0 |  | 08107 | 0 |  | 08049 | 0 |
| 08520 | 0.043604796 |  | 19118 | 0 |  | 08108 | 0 |  | 08050 | 0 |
| 19114 | 0.040358404 |  | 19525 | 0 |  | 08201 | 0 |  | 08051 | 0 |
| 08701 | 0.037428835 |  | 08031 | 0 |  | 08203 | 0 |  | 08053 | 0 |
| 08109 | 0.035426874 |  | 08046 | 0 |  | 08205 | 0 |  |  |  |
| 18901 | 0.034600817 |  | 08054 | 0 |  | 19435 | 0 |  |  |  |
| 08628 | 0.034449978 |  | 08073 | 0 |  | 19436 | 0 |  |  |  |
