# Supplementary material for: Environmental exposomics and lung cancer risk assessment in the Philadelphia metropolitan area using ZIP code–level hazard indices
Source: Environ Sci Pollut Res Int. Author manuscript; Available in PMC 2021 Jul 2. (PMC8238722; doi:10.1007/s11356-021-12884-z)
Supplement: Supplemental Table 4 [file NIHMS1676090-supplement-Supplemental_Table_4.docx]

**Supplemental Table 4.** Total air emissions from 201 selected chemicals for ZIP codes in the study area.

| **ZIP code** | **Air Emissions (lbs)** |  | **ZIP code** | **Air Emissions (lbs)** |  | **ZIP code** | **Air Emissions (lbs)** |  | **ZIP code** | **Air Emissions (lbs)** |
| --- | --- | --- | --- | --- | --- | --- | --- | --- | --- | --- |
| 19007 | 24558902.8 |  | 18936 | 155719.5601 |  | 19128 | 0 |  | 19465 | 0 |
| 19310 | 23538707 |  | 19090 | 155697 |  | 19138 | 0 |  | 08217 | 0 |
| 19061 | 19652500.77 |  | 08094 | 148758.714 |  | 19139 | 0 |  | 08221 | 0 |
| 19154 | 16285211.28 |  | 19702 | 140706.64 |  | 19141 | 0 |  | 08225 | 0 |
| 19137 | 15945469.04 |  | 08344 | 140237 |  | 19147 | 0 |  | 19472 | 0 |
| 19426 | 13006385 |  | 19701 | 140124.0057 |  | 08097 | 0 |  | 19473 | 0 |
| 19713 | 10744886.65 |  | 19114 | 122030.0121 |  | 19149 | 0 |  | 19475 | 0 |
| 19804 | 10294760.62 |  | 19142 | 115553 |  | 19150 | 0 |  | 19492 | 0 |
| 19145 | 9321891.093 |  | 19153 | 113570.46 |  | 19151 | 0 |  | 08232 | 0 |
| 19706 | 8842666.649 |  | 08312 | 110285 |  | 19152 | 0 |  | 08241 | 0 |
| 19134 | 8295961.683 |  | 19029 | 108447 |  | 19803 | 0 |  | 19525 | 0 |
| 08093 | 6933739.813 |  | 19390 | 104530 |  | 18902 | 0 |  | 08244 | 0 |
| 18951 | 6874586.153 |  | 08048 | 91238.108 |  | 18912 | 0 |  | 08317 | 0 |
| 19720 | 4388569.939 |  | 18964 | 89638 |  | 18913 | 0 |  | 08319 | 0 |
| 19454 | 3693942 |  | 19438 | 88141.6362 |  | 18917 | 0 |  | 08322 | 0 |
| 19464 | 3451641.964 |  | 08561 | 86086 |  | 18920 | 0 |  | 08326 | 0 |
| 08066 | 3344306.571 |  | 08081 | 81529 |  | 18923 | 0 |  | 08328 | 0 |
| 08027 | 2897382.68 |  | 08609 | 78059 |  | 18925 | 0 |  | 19707 | 0 |
| 08109 | 2634864 |  | 19115 | 77818 |  | 18929 | 0 |  | 19710 | 0 |
| 08016 | 2544962.944 |  | 08691 | 71076 |  | 18930 | 0 |  | 19716 | 0 |
| 19446 | 2427790.12 |  | 08052 | 70031.58 |  | 19805 | 0 |  | 19717 | 0 |
| 08110 | 2355205.6 |  | 18976 | 67782.92 |  | 19807 | 0 |  | 19730 | 0 |
| 19403 | 2334597 |  | 19131 | 65920 |  | 19810 | 0 |  | 19731 | 0 |
| 19130 | 2333027 |  | 19023 | 65800 |  | 18932 | 0 |  | 19732 | 0 |
| 08014 | 2103447.681 |  | 08505 | 65551.04 |  | 18935 | 0 |  | 19733 | 0 |
| 19335 | 2078420.35 |  | 08240 | 60842 |  | 18938 | 0 |  | 19734 | 0 |
| 19382 | 2004509 |  | 19341 | 52935.60766 |  | 18942 | 0 |  | 19735 | 0 |
| 19355 | 1969257.142 |  | 19144 | 46751 |  | 18947 | 0 |  | 19736 | 0 |
| 08011 | 1829409.881 |  | 19143 | 44312.22 |  | 18950 | 0 |  | 19072 | 0 |
| 19078 | 1693815 |  | 08690 | 43152 |  | 18954 | 0 |  | 19074 | 0 |
| 18944 | 1526376.79 |  | 19008 | 43000 |  | 18955 | 0 |  | 19075 | 0 |
| 19136 | 1397956.006 |  | 19135 | 41907.82324 |  | 18962 | 0 |  | 19076 | 0 |
| 08224 | 1257237.1 |  | 08060 | 41000 |  | 18970 | 0 |  | 19079 | 0 |
| 19020 | 1200053 |  | 08002 | 37639 |  | 18972 | 0 |  | 19081 | 0 |
| 18969 | 1171939.51 |  | 19442 | 36309.37 |  | 18977 | 0 |  | 19082 | 0 |
| 18960 | 1167965.011 |  | 19140 | 35930.194 |  | 18980 | 0 |  | 19083 | 0 |
| 08611 | 1162296.793 |  | 08007 | 33211 |  | 19001 | 0 |  | 19085 | 0 |
| 08215 | 1119927.9 |  | 19350 | 33170.104 |  | 19003 | 0 |  | 19086 | 0 |
| 08009 | 1112030.523 |  | 08104 | 28742.57 |  | 19009 | 0 |  | 19094 | 0 |
| 08628 | 1082023 |  | 19808 | 26763 |  | 19010 | 0 |  | 19095 | 0 |
| 08619 | 1076982 |  | 08056 | 25361 |  | 19012 | 0 |  | 19096 | 0 |
| 19013 | 1071510.623 |  | 08638 | 23707.68 |  | 19017 | 0 |  | 08341 | 0 |
| 18041 | 1048396.323 |  | 08753 | 22792 |  | 19025 | 0 |  | 08346 | 0 |
| 19067 | 1039290.722 |  | 08550 | 20900 |  | 19026 | 0 |  | 08350 | 0 |
| 08518 | 1016017.76 |  | 19401 | 20664.31 |  | 19027 | 0 |  | 08401 | 0 |
| 18974 | 971758.2253 |  | 19064 | 17411.7 |  | 19031 | 0 |  | 08402 | 0 |
| 19022 | 966752.4343 |  | 19073 | 16960.1 |  | 19033 | 0 |  | 08403 | 0 |
| 08648 | 950181 |  | 19015 | 16960 |  | 19035 | 0 |  | 08406 | 0 |
| 19709 | 914674.2 |  | 08310 | 14200 |  | 19036 | 0 |  | 08515 | 0 |
| 08096 | 912855 |  | 08733 | 14018.83 |  | 19041 | 0 |  | 08525 | 0 |
| 08057 | 902679.6419 |  | 08103 | 13594.24098 |  | 19043 | 0 |  | 08527 | 0 |
| 19030 | 876319.95 |  | 19124 | 12909.248 |  | 19054 | 0 |  | 08540 | 0 |
| 18901 | 833720.004 |  | 19087 | 12034 |  | 19055 | 0 |  | 08542 | 0 |
| 18940 | 817459.836 |  | 19129 | 11808.07461 |  | 19056 | 0 |  | 08004 | 0 |
| 19428 | 806738.38 |  | 19123 | 10643 |  | 19060 | 0 |  | 08005 | 0 |
| 19112 | 723147.323 |  | 18070 | 10581.51 |  | 19063 | 0 |  | 08006 | 0 |
| 19046 | 718513.14 |  | 19032 | 9871 |  | 19066 | 0 |  | 08008 | 0 |
| 19044 | 718029 |  | 19468 | 7849.95 |  | 19070 | 0 |  | 08010 | 0 |
| 08028 | 714893 |  | 08055 | 7587 |  | 08054 | 0 |  | 08015 | 0 |
| 19116 | 679404.3181 |  | 18915 | 6718 |  | 08059 | 0 |  | 08019 | 0 |
| 19348 | 652297 |  | 08618 | 6250 |  | 08061 | 0 |  | 08554 | 0 |
| 18966 | 630897.11 |  | 19358 | 6244.0719 |  | 08062 | 0 |  | 08560 | 0 |
| 19380 | 595349.555 |  | 08533 | 6134 |  | 08063 | 0 |  | 08562 | 0 |
| 19440 | 560975.9374 |  | 19520 | 5694.850357 |  | 08064 | 0 |  | 08610 | 0 |
| 19801 | 551934.6357 |  | 08620 | 5410.6 |  | 08068 | 0 |  | 08629 | 0 |
| 08086 | 546730.4577 |  | 19125 | 5114.03119 |  | 08073 | 0 |  | 08640 | 0 |
| 19460 | 536919.5304 |  | 19121 | 5030 |  | 08074 | 0 |  | 18054 | 0 |
| 19148 | 509134.3 |  | 08641 | 3961.0003 |  | 08078 | 0 |  | 08721 | 0 |
| 19474 | 503684 |  | 08759 | 3825 |  | 08080 | 0 |  | 08722 | 0 |
| 19703 | 495707.114 |  | 08608 | 3335.6 |  | 08083 | 0 |  | 08723 | 0 |
| 19457 | 458046 |  | 19711 | 3300.44 |  | 08084 | 0 |  | 08731 | 0 |
| 19002 | 438545 |  | 19021 | 3193.819989 |  | 08087 | 0 |  | 08732 | 0 |
| 08085 | 429763.1683 |  | 19133 | 2500 |  | 08089 | 0 |  | 08734 | 0 |
| 18073 | 427412 |  | 19127 | 2367.563201 |  | 08090 | 0 |  | 08735 | 0 |
| 08102 | 427295 |  | 08036 | 1858 |  | 08091 | 0 |  | 08738 | 0 |
| 19034 | 423907 |  | 19111 | 1600 |  | 08092 | 0 |  | 08740 | 0 |
| 08701 | 423329.32 |  | 19363 | 1600 |  | 08095 | 0 |  | 18074 | 0 |
| 19057 | 412758.252 |  | 19004 | 1450 |  | 19312 | 0 |  | 18076 | 0 |
| 19050 | 399394 |  | 08105 | 924 |  | 19316 | 0 |  | 18077 | 0 |
| 19014 | 365223.6757 |  | 08234 | 819.73 |  | 19317 | 0 |  | 18081 | 0 |
| 18914 | 356729 |  | 19806 | 750 |  | 19319 | 0 |  | 08020 | 0 |
| 19405 | 350916 |  | 19038 | 500 |  | 19333 | 0 |  | 08021 | 0 |
| 19132 | 349257.96 |  | 19047 | 463 |  | 19342 | 0 |  | 08022 | 0 |
| 08030 | 330297.4603 |  | 19422 | 418 |  | 19343 | 0 |  | 08029 | 0 |
| 19146 | 314915.4561 |  | 19106 | 316 |  | 19345 | 0 |  | 08031 | 0 |
| 08071 | 307365 |  | 08534 | 295 |  | 19352 | 0 |  | 08033 | 0 |
| 08330 | 291700 |  | 08075 | 273 |  | 19362 | 0 |  | 08741 | 0 |
| 19344 | 281360 |  | 19053 | 83 |  | 19365 | 0 |  | 08742 | 0 |
| 08034 | 281011 |  | 08755 | 10 |  | 19367 | 0 |  | 08751 | 0 |
| 19809 | 275410.426 |  | 08088 | 8 |  | 19373 | 0 |  | 08752 | 0 |
| 08520 | 268621 |  | 08511 | 2.74 |  | 19374 | 0 |  | 08757 | 0 |
| 19018 | 263584 |  | 08012 | 2.076 |  | 19375 | 0 |  | 08758 | 0 |
| 08026 | 234000 |  | 19107 | 1.000566587 |  | 19383 | 0 |  | 08035 | 0 |
| 19120 | 224435.754 |  | 19372 | 1 |  | 19425 | 0 |  | 08039 | 0 |
| 08003 | 219264.15 |  | 08724 | 0.1 |  | 08106 | 0 |  | 08041 | 0 |
| 08037 | 215306 |  | 19330 | 0.01 |  | 08107 | 0 |  | 08042 | 0 |
| 19301 | 204315 |  | 19311 | 0.0023251 |  | 08108 | 0 |  | 08043 | 0 |
| 19406 | 197498.5 |  | 19102 | 0 |  | 08201 | 0 |  | 08045 | 0 |
| 19040 | 197497.32 |  | 19103 | 0 |  | 08203 | 0 |  | 08046 | 0 |
| 19006 | 197267.16 |  | 19104 | 0 |  | 08205 | 0 |  | 08049 | 0 |
| 19477 | 191690 |  | 19109 | 0 |  | 19435 | 0 |  | 08050 | 0 |
| 19320 | 179370.19 |  | 19113 | 0 |  | 19436 | 0 |  | 08051 | 0 |
| 08065 | 174027 |  | 19118 | 0 |  | 19437 | 0 |  | 08053 | 0 |
| 08077 | 168008.1 |  | 19119 | 0 |  | 19444 | 0 |  |  |  |
| 19462 | 161550 |  | 19122 | 0 |  | 19453 | 0 |  |  |  |
| 19802 | 161300 |  | 19126 | 0 |  | 19456 | 0 |  |  |  |
